# Role of Robotic Surgery and Artificial Intelligence in the Management of Abdominal Wall Hernias: A Systematic Review

**DOI:** 10.7759/cureus.99354

**Published:** 2025-12-16

**Authors:** Dimitrios Bakaoukas, Natalia Sinou, Nikoleta Sinou, Athanassios Marinis, Dimitrios Filippou

**Affiliations:** 1 Department of Surgery, Tzaneio Prefecture General Hospital of Piraeus, Piraeus, GRC; 2 Research and Education Institute in Biomedical Sciences, National and Kapodistrian University of Athens School of Medicine, Athens, GRC; 3 1st Department of General Surgery, Tzaneio Prefecture General Hospital of Piraeus, Piraeus, GRC; 4 Department of Anatomy, National and Kapodistrian University of Athens School of Medicine, Athens, GRC

**Keywords:** abdominal wall, artificial intelligence, hernias, robotic, surgery

## Abstract

Abdominal wall hernias impact millions of individuals annually. Two rapidly advancing technologies that can significantly enhance hernia management are robotic surgery, with its ergonomic features and technical benefits, and artificial intelligence, renowned for its groundbreaking computational capabilities. Robotic surgery appears to provide numerous advantages for treating both inguinal and ventral hernias, while artificial intelligence has been incorporated in various applications, indicating its potential to improve hernia repair outcomes. Nevertheless, both technologies face several challenges and limitations.

A systematic review was carried out using the PubMed and Scopus databases with the search terms "robotic" AND "abdominal wall hernias" AND "artificial intelligence", resulting in 46 articles reviewed.

This article examines the pros and cons of robotic surgery, explores the potential applications of artificial intelligence, and discusses the hurdles both technologies encounter. Ultimately, it concludes that although these innovations present many benefits, further high-quality research is essential, along with overcoming numerous barriers, for their effective integration into hernia repair procedures.

## Introduction and background

Abdominal wall hernias rank among the most prevalent surgical issues worldwide. Inguinal hernias make up about 75% of all hernias, with estimates indicating that over 20 million repairs occur annually. Ventral hernias are also common, with incisional hernias appearing in 10% of post-surgical patients and umbilical hernias affecting 5% of the general population [1,2].

Hernia repair methods are diverse, featuring a range of surgical approaches and techniques. Traditionally, during the 20th century, repairs were performed using an open technique. Methods like Shouldice, Bassini, and later Lichtenstein allowed for the effective repair of inguinal hernias at a low cost and typically without general anesthesia. The laparoscopic method emerged in the 1990s for treating inguinal hernias, bringing the advantages of minimally invasive surgery (MIS) into hernia repair [3,4].

In today's age of technological advancements, robotic surgery is increasingly being incorporated. It offers various ergonomic and technical benefits, such as three-dimensional visualization and seven degrees of movement, rivaling the laparoscopic approach and potentially enhancing care for hernia patients [5].

Artificial intelligence (AI) represents another groundbreaking development that could transform hernia repair. With machine learning (ML) capabilities and substantial computational power, AI has the potential to improve hernia management across multiple stages, including diagnosis, prognosis, and the surgical procedure itself [6].

## Review

Materials and methods

**Figure 1:**
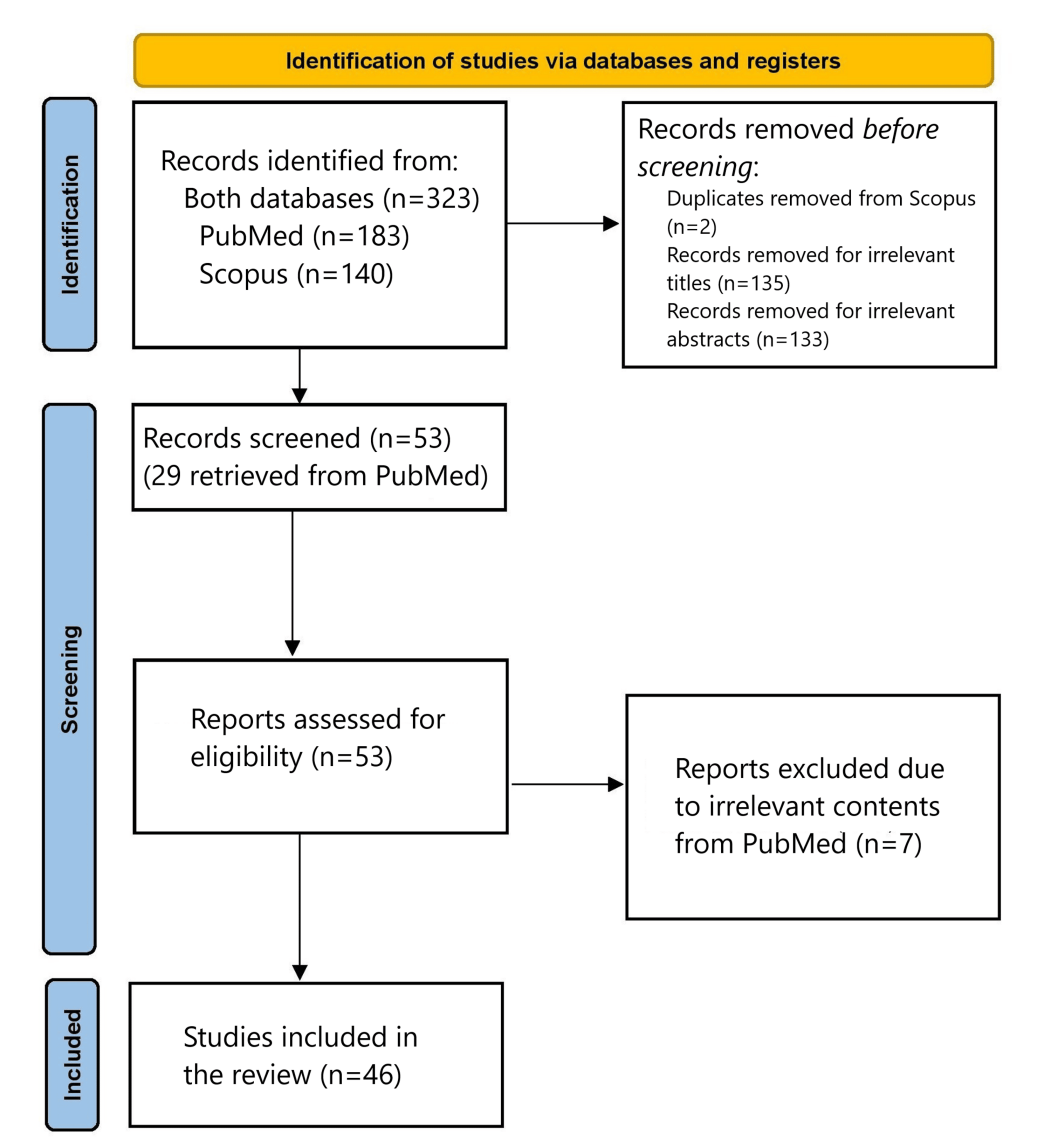
PRISMA 2020 flow diagram for new systematic reviews which included searches of databases and registers only PRISMA: Preferred Reporting Items for Systematic Reviews and Meta-Analyses

A comprehensive investigation was performed using published literature sourced from the PubMed and Scopus databases. The search terms employed were "robotic" AND "abdominal wall hernias" AND "artificial intelligence". The study adhered to the Preferred Reporting Items for Systematic Reviews and Meta-Analyses (PRISMA) 2020 flow diagram guidelines for new systematic reviews, which included searching through databases, registries, and other resources (Figure 1). Specifically, from the initial search, 323 articles were identified, 183 coming from PubMed and 140 from Scopus. Before screening, two duplicate records were removed from the Scopus database, 135 were removed due to irrelevant titles, and 133 were removed due to irrelevant abstracts. Fifty-three articles were screened, 29 of which were retrieved from similar articles from the PubMed database. Among the 53 articles, seven were excluded from the PubMed database due to irrelevant content. Consequently, a total of 46 studies were utilized.

Results

Among the 46 articles analyzed, 29 (63%) concentrated on robotic surgery, 16 (35%) on AI, and one (2%) addressed both topics. This selection included 14 (30%) reviews, six randomized controlled trials (13%), seven cohort studies (15%), and 12 observational studies (26%), along with three case series, one case-control study, one case report, one meta-analysis, and one quality improvement research. A total of 23 studies emphasized the benefits of robotic surgery, with 16 discussing inguinal hernias, 10 focusing on ventral hernias, six examining additional benefits of the robotic platform, eight outlining the limitations of the robot, 17 exploring potential applications of AI, and four addressing AI's limitations (Table 1).

**Table 1:** Studies included and the main topics they are referring to AI: artificial intelligence; RCT: randomized controlled trial

	Advantages of robotic surgery	Inguinal hernia	Ventral hernia	Other advantages	Limitations of robotic surgery	Applications of AI	Limitations of AI	Type of study
Helgstrand et al., 2024 [1]	Yes	No	No	No	Yes	No	No	Review
Peltrini et al., 2023 [2]	Yes	Yes	No	No	No	No	No	Multicenter retrospective
Ramser et al., 2021 [3]	Yes	Yes	No	No	Yes	No	No	Video report and case series
Morrell et al., 2021 [4]	Yes	Yes	No	No	Yes	No	No	Retrospective chart review
Lomanto et al., 2024 [5]	Yes	Yes	Yes	Yes	Yes	No	No	Review
Vogel and Mück, 2024 [6]	No	No	No	No	No	Yes	Yes	Review
Halabi et al., 2024 [7]	No	No	No	No	No	No	No	Retrospective chart review
Donkor et al., 2017 [8]	Yes	Yes	Yes	No	No	No	No	Review
Anoldo et al., 2024 [9]	Yes	Yes	Yes	No	No	No	No	Review
Li et al., 2024 [10]	Yes	Yes	No	No	No	No	No	Meta-analysis
Biswas et al., 2023 [11]	Yes	Yes	No	No	No	No	No	Review
Valorenzos et al., 2024 [12]	Yes	Yes	No	No	No	No	No	Prospective RCT
Andreou et al., 2023 [13]	Yes	Yes	No	No	No	No	No	Review and cohort
Chao et al., 2024 [14]	Yes	Yes	No	No	No	No	No	Retrospective observational
Okamoto et al., 2023 [15]	No	Yes	No	No	No	No	No	Retrospective cohort
Lade et al., 2023 [16]	Yes	Yes	No	No	No	No	No	Review
Prabhu et al., 2020 [17]	Yes	Yes	No	No	No	No	No	RCT
Marckmann et al., 2025 [18]	Yes	No	Yes	No	No	No	No	Cohort
Henriksen et al., 2024 [19]	Yes	No	Yes	No	Yes	No	No	Retrospective cohort
Warren et al., 2024 [20]	Yes	No	Yes	No	No	No	No	Prospective RCT
Dhanani et al., 2023 [21]	No	No	Yes	No	No	No	No	RCT
Petro et al., 2021 [22]	Yes	No	Yes	No	Yes	No	No	RCT
Olavarria et al., 2020 [23]	No	No	Yes	No	No	No	No	RCT
Dewulf et al., 2022 [24]	No	No	No	Yes	No	No	No	Case series and review
Lima et al., 2023 [25]	No	No	No	Yes	No	No	No	Case report
Di Giuseppe et al., 2020 [26]	No	No	No	Yes	No	No	No	Case series
Lunardi et al., 2024 [27]	Yes	No	No	Yes	No	No	No	Cohort
Amaral et al., 2022 [28]	Yes	Yes	No	Yes	No	No	No	Cohort
Sabbatini et al., 2024 [29]	Yes	No	Yes	No	Yes	No	No	Observational
Choi et al., 2023 [30]	Yes	Yes	No	No	Yes	No	No	Retrospective observational
Elhage et al., 2021 [31]	No	No	No	No	No	Yes	No	Quality improvement
Taha et al., 2022 [32]	No	No	No	No	No	Yes	Yes	Review
Dai et al., 2024 [33]	No	No	No	No	No	Yes	No	Case-control
Yu et al., 2025 [34]	No	No	No	No	No	Yes	No	Retrospective observational
Dong et al., 2024 [35]	No	No	No	No	No	Yes	No	Retrospective observational
Kitaguchi et al., 2022 [36]	No	No	No	No	No	Yes	Yes	Review
Zygomalas et al., 2024 [37]	No	No	No	No	No	Yes	No	Prospective observational
Takeuchi et al., 2023 [38]	No	No	No	No	No	Yes	No	Observational
Cui et al., 2021 [39]	No	No	No	No	No	Yes	No	Retrospective observational
Ortenzi et al., 2023 [40]	No	No	No	No	No	Yes	No	Observational
Choksi et al., 2023 [41]	No	No	No	No	No	Yes	No	Observational
Zang et al., 2023 [42]	No	No	No	No	No	Yes	No	Observational
Takeuchi et al., 2022 [43]	No	No	No	No	No	Yes	No	Observational
Shin et al., 2024 [44]	No	No	No	No	No	Yes	No	Prospective cohort
Riddle et al., 2024 [45]	No	No	No	No	No	Yes	No	Review
Iftikhar et al., 2024 [46]	Yes	No	No	No	No	Yes	Yes	Review

Discussion

General Advantages of Robotic Surgery

In recent years, hernia surgeries utilizing robotic systems like the da Vinci and Versius (CMR Surgical, Cambridge, United Kingdom) have demonstrated safety and are increasingly favored, largely due to the multitude of advantages they provide [7,8]. The robotic technique falls under the category of MIS, resulting in diminished wound complications, quicker recovery times, improved pain management, smaller incisions, and reduced tissue damage [9-11]. It offers technical and ergonomic benefits that lessen surgeon tiredness, such as wristed movements, three-dimensional visualization, and the ability to counteract tremors and the fulcrum effect commonly seen in laparoscopy [5]. These attributes lead to increased precision in handling tissue, dissection, suturing, fascial defect closure, mesh placement, and reduced neurovascular injury [5,12]. Enhanced visibility of the surgical field allows for more thorough evaluation of anatomical structures, promoting a more anatomy-focused method for hernia repair [13]. This visualization is particularly advantageous in obese patients, as the robotic ports counterbalance the weight of the abdominal wall, permitting lower intra-abdominal pressure in individuals with cardiopulmonary issues [3]. Additionally, it presents numerous educational opportunities, especially when a dual console is employed [3].

Inguinal Hernias

The most prevalent techniques for inguinal hernia repair are robotic transabdominal preperitoneal (rTAPP) and robotic totally extraperitoneal (rTEP). Generally, the robotic method is considered superior to the open approach, as it is associated with fewer complications, reduced blood loss, less postoperative pain, lower rates of reoperations, and shorter hospital stays [3,5,8]. When compared to the laparoscopic method, it shows similar outcomes, with some studies indicating benefits such as reduced recurrence rates, fewer conversions to open surgery, decreased postoperative pain, less opioid consumption, shorter dissection time for medial hernias, and advantages in more complicated cases [3,5,9,14,15]. However, certain studies did not demonstrate its superiority and pointed out drawbacks like increased surgeon frustration, higher costs, and longer operation times associated with the robotic method [2,10,17]. Additionally, it is deemed safe for patients who have previously undergone prostatectomy, a limitation for the laparoscopic technique [16]. Furthermore, Andreou et al. [13] noted that the quality of robotic vision is so exceptional that it facilitates an anatomy-guided hernia repair by enabling the identification of Hesselbach's ligament.

Ventral Hernias

The robotic platform stands out when compared to both open and laparoscopic techniques by enabling access to the preperitoneal and retromuscular spaces, thus allowing procedures like r-ventral-TAPP and robotic transabdominal umbilical prosthetic (rTARUP). These methods are associated with fewer wound complications, reduced chronic pain, and a lower requirement for mesh fixation compared to the intraperitoneal onlay mesh (IPOM) technique [5,18]. Numerous studies have indicated that the robotic method is superior to the open technique, resulting in shorter hospital stays and fewer readmissions, reoperations, complications, blood transfusions, and recurrence rates [5,18,19]. However, Warren et al. [20] did not find a significant advantage for the robotic approach concerning outcomes, while Henriksen et al. [19] suggested that when considering reduced readmissions and reoperations, the robot may be more cost-effective than the open method. In comparison to the laparoscopic technique, the robotic approach is linked to fewer conversions to open surgery, lower recurrence and reoperation rates, shorter hospital stays, less postoperative pain, and improved fascial closure, which minimizes recurrence risks. Furthermore, the robotic system allows for transversus abdominis release (TAR), which is essential for closing large hernia defects, a procedure not achievable through laparoscopy [5,18,21]. Yet, research by Petro et al. [22] and Olavarria et al. [23] demonstrated no differences in patient outcomes alongside higher costs and longer operative times for robotic surgery. Henriksen et al. [19] argue that primary ventral hernias are generally simpler to repair via the open approach, providing better cost efficiency. The benefits of robotic surgery appear to be more pronounced in complex ventral and incisional hernias, where they show advantageous outcomes and cost-effectiveness.

Other Advantages, Challenges, and Limitations of Robotic Surgery

The robotic platform has been effectively used to treat parastomal hernias (PH), which are often intricate and challenging to manage. It has enabled the implementation of numerous techniques that enhance PH repair [24]. Additionally, safe repairs of Spigelian and flank hernias have been achieved [25,26]. Lunardi et al. [27] demonstrated that robotic surgery is applicable for various urgent general surgery conditions, including strangulated inguinal and ventral hernias, showcasing fewer conversions to open surgery and reduced length of stay compared to laparoscopic methods. Amaral et al. [28] successfully employed robotics to address recurrent hernias following laparoscopic repairs.

One of the primary drawbacks of the robotic platform, highlighted in multiple studies, is its higher cost relative to other techniques [2,17,22,23]. However, research by Henriksen et al. [19] and Sabbatini et al. [29] reveals that when comprehensive costs are assessed, including shorter hospital stays, medical salaries, and reduced readmissions and reoperations, expenses can align with those of open surgery. Another issue is the extended operative time associated with robotic surgery when compared to open and laparoscopic techniques. As noted by Morrell et al. [4] and Anoldo et al. [9], this may stem from the docking process of the robotic system and the surgical team's lack of experience. As familiarity with the robotic platform increases and technology advances, operating times are expected to decrease. Furthermore, robotic surgery presents a steep learning curve, requiring a specialized training framework and high procedural volume to optimize outcomes [1,3,5]. Nonetheless, Choi et al. [30] reported a shorter learning curve for experienced laparoscopic surgeons operating on inguinal hernias. Additionally, further research is necessary to evaluate the long-term results of robotic hernia repair, with randomized controlled trials essential to assess the efficacy of this method against others [5]. Lastly, the limited availability of robotic platforms in many countries constrains their global application [5].

*AI* 

AI represents one of the most swiftly evolving technologies of the 21st century. It encompasses various subfields, including ML methods, which perform diverse tasks using existing data without further human guidance. Deep learning methods (DLM), a subset of ML, employ artificial neural networks that mimic the brain's neural structures to adapt to new information, develop, and tackle complex problems. In the domain of computer vision (CV), AI is utilized to analyze images and videos. These capabilities, along with AI's ability to surpass human cognitive limits sans human error, highlight its potential to revolutionize hernia management [6,31,32].

Potential Roles of AI in Hernia Repair 

Elhage et al. [31] applied DLM to assess CT images, accurately predicting abdominal wall reconstruction complexity at levels exceeding those of expert professionals. Beyond these findings, other studies have effectively predicted post-surgical complications, particularly surgical site infections, hernia recurrence, and 30-day readmission rates for both inguinal and ventral hernias [6,31,32]. For incisional hernias, ML models have been implemented to guide patients and surgeons, gauge tissue elasticity, identify incisional hernia recurrence risk factors, and predict recurrences with success [32]. These AI capabilities could assist in the risk assessment of hernia patients, guiding the surgical approach, decision-making, and patient consent [31]. Furthermore, Dai et al. [33] created an ML model to forecast PH occurrences in individuals undergoing permanent colostomy post-colorectal cancer treatment, while Yu et al. [34] devised a model to predict inguinal hernia incidence within a year following robot-assisted radical prostatectomy. Dong et al. [35] utilized an image-based DL model to efficiently predict hernia occurrence in patients with temporary ileostomy.

From another angle, AI can enhance the surgical process itself and be integrated into MIS. MIS, particularly robotic surgery, can generate high-quality surgical data, images, and videos essential for creating robust AI algorithms that provide anatomical insights, aid surgeons in complex cases, deliver real-time decision support, and improve patient care [36]. AI has demonstrated proficiency in identifying anatomical structures during MIS. Studies by Zygomalas et al. [37] and Takeuchi et al. [38] confirmed that AI could pinpoint critical anatomical landmarks and surgical instruments during laparoscopic TAPP inguinal hernia repair. Similarly, Cui et al. [39] used a convolutional neural network, a kind of DLM, to accurately identify the vas deferens during laparoscopic inguinal hernia repair, addressing a frequent complication. A promising AI application could involve fluorescence imaging, enabling the identification of concealed structures, such as blood vessels and nerves [6]. These capabilities could provide anatomical guidance to surgeons and help mitigate complications. Another significant implementation of AI technology involves surgical phase recognition. Numerous studies have validated AI's effectiveness in automatically identifying various surgical stages, primarily in laparoscopic TAPP and TEP for inguinal hernia repair. This automation enhances data extraction, workflow analysis, and efficiency optimization of surgical teams [40-43]. Additionally, Shin et al. [44] utilized ML models for robotic ventral and incisional hernia repairs to predict postoperative outcomes and complications based not only on patient characteristics but also on measurable performance indicators, including camera control and other operator-dependent metrics. AI can also support training for hernia repairs by helping formulate training programs and effectively gauging trainees' levels based on video analyses [32,45]. Finally, though its use in hernia management is currently limited, AI can provide vital decision support for surgeons during incisional hernia repair [32].

Challenges and Limitations of AI 

Given the restricted data on AI applications in hernia management, further investigation into this subject is required. Additionally, AI brings forth multiple ethical concerns about accountability in case of complications, patient privacy, and the dynamics between patients and AI systems. Challenges also include cybersecurity threats, where hacking may jeopardize patient safety, along with increased costs and resource demands for AI implementation in hernia repair, as well as the necessity for high-quality data to train AI algorithms [36,46].

## Conclusions

Robotic surgery and AI are groundbreaking technologies that present numerous advantages and may advance hernia management significantly. Robotic surgery exhibits promising results, and it could offer more benefits to patients compared to the traditional open and laparoscopic approaches. Nonetheless, further investigations, particularly randomized controlled trials, are essential to confirm the effectiveness of robotic systems definitively. On the other hand, it has been shown that AI can be a valuable tool that could reshape the way hernias are approached and treated. However, additional research must focus on the role of AI in treating abdominal wall hernias, and various obstacles must be addressed for these innovations to be successfully integrated into practice.
